# Novel *PLA2G6* Pathogenic Variants in Chinese Patients With *PLA2G6*-Associated Neurodegeneration

**DOI:** 10.3389/fneur.2022.922528

**Published:** 2022-07-13

**Authors:** Yalan Wan, Yanyan Jiang, Zhiying Xie, Chen Ling, Kang Du, Ran Li, Yun Yuan, Zhaoxia Wang, Wei Sun, Haiqiang Jin

**Affiliations:** ^1^Department of Neurology, Peking University First Hospital, Beijing, China; ^2^Department of Neurology, The First Affiliated Hospital of Zhengzhou University, Zhengzhou, China; ^3^Department of Neurology, Huoguosi TCM Hospital Affiliated to Beijing University of Chinese Medicine, Beijing, China

**Keywords:** *PLA2G6* gene, iron deposition, atypical neuroaxonal dystrophy, parkinsonism, neurogenetic

## Abstract

**Background:**

*PLA2G6*-associated neurodegeneration (PLAN) is a heterogeneous group of neurodegenerative diseases caused by biallelic *PLA2G6* mutations, covering diseases such as infantile neuroaxonal dystrophy (INAD), atypical neuroaxonal dystrophy (ANAD), dystonia parkinsonism (DP), and autosomal recessive early-onset parkinsonism (AREP). The study aims to report the clinical and genetic features of a series of PLAN patients.

**Methods:**

The clinical and radiological findings of five Chinese patients from three families were collected. Whole-exome next generation sequencing (NGS) was applied to identify the genetic causes. Co-segregation analysis of the detected candidate variants were performed in their families. The pathogenicity of identified novel variants was predicted by *in silico* analysis.

**Results:**

NGS revealed compound heterozygous variants of *PLA2G6* gene in all five patients. There were six *PLA2G6* variants identified, including two known variants (c.116G>A, c.238G>A) and four novel variants (c.2120dupA, c.2071C>G, c.967G>A, c1534T>A). ACMG predicts c.2120dupA to be pathogenic, c.2071C>G and c.1534T>A to be likely pathogenic, and c1534T>A to be of uncertain significance. Clinically, four patients fell into the diagnosis of ANAD, and 1 into the diagnosis of AREP. Brain imaging revealed cerebellar atrophy, iron deposition in bilateral globus pallidus, and substantia nigra in three cases.

**Conclusions:**

Four novel pathogenic variants were discovered and the pathogenic variant spectrum of the *PLA2G6* gene was expanded.

## Introduction

The *PLA2G6* gene was initially cloned in two unrelated Israeli infantile neuroaxonal dystrophy (INAD) families in 2006 (1). Later *PLA2G6* pathogenic variants were identified in atypical neuroaxonal dystrophy (ANAD) and parkinsonian syndrome including adult onset dystonia parkinsonism (DP) and autosomal recessive early-onset parkinsonism (AREP) (2). Thus *PLA2G6*-associated neurodegeneration (PLAN) appears as a heterogeneous group of neurodegenerative diseases including INAD, ANAD, DP, and AREP.

INAD, first described by Seitelberger in 1952, emerges with developmental regression, hypotonia, and spastic tetraparesis, with an onset between 6 months and 3 years (3). ANAD, first described by Nardocci et al. (4) in 1999, is of a milder form compared with INAD, with a later onset (up to 6 years) and a more protracted course (4). The main symptoms of ANAD include cerebellar signs, gait abnormalities, speech delay or regression, and diminished social interaction which may lead to a misdiagnosis of autism before the occurrence of other neurological signs (5). In 2009, Paisa'n-Ruiz et al. (6) identified *PLA2G6* pathogenic variants in patients suffering from early-onset levodopa responsive dystonia-parkinsonism and broadened the phenotypic spectrum of PLAN. The phenotype of AREP, first reported by Shi CH, et al. (7) in 2011, is characterized by extrapyramidal signs, cognitive decline, dystonia, dysarthria/dysphonia, swallowing problems, limb tremors, and abnormal gait, sensitive to dopaminergic agents. In recent years, considering common features of DP, AREP, and sporadic early onset parkinsonism (EOP), scholars have proposed the concept of phenotypic continuum to describe *PLA2G6*-related parkinsonism, the most common phenotype in late-onset PLAN (8, 9).

PLAN has been reported worldwide, but is still not a commonly detected disease. Up to now, there are only 21 ANAD and 86 *PLA2G6*-related parkinsonism patients (8) reported in literature. Herein this study reported the clinical features and PLAN pathogenic variants in 4 Chinese patients with ANAD and 1 with AREP (Table 1), carrying 6 different mutations, 4 of which are novel. Furthermore, the reported ANAD and adult onset AREP in literature were reviewed, with their clinical features further analyzed.

**Table 1:** Clinical features of the five PLAN patients in this article.

**Case**	**Pedigree**	**Gender/ Age at Onset**	**Ethnicity**	**Presenting Symptom**	**Pyramidal signs**	**Hypertonia**	**Nystagmus / Strabismus**	**Other signs**	**Axonal spheroids**	**Cerebellar atrophy**	**Iron deposition in basal ganglia**	**Genotype**	**Transcript ID**
1	A–II 1	M/14yr	Chinese	Walk unstably	+	Yes–lower limbs	–	Cerebellar signs, dysarthria, and intellectual disability	ND	+	+	Compound heterozygous c.2120dupA and c.2071C>G	NM_001004426
2	A–II 2	M/8yr	Chinese	Run more slowly	+	Yes–lower limbs	–	Cerebellar signs	–	+	+	Compound heterozygous c.2120dupA and c.2071C>G	NM_001004426
3	B–II 1	F/3yr	Chinese	Weakness and rigidity in lower limbs	Yes–left	Yes–lower limbs	–	intellectual disability	ND	+	+	Compound heterozygous c.238G>A and c.1534T>A	NM_003560
4	B–II 2	F/2yr	Chinese	Have difficulties walking	+	Yes–lower limbs	–	intellectual disability	–	+	–	Compound heterozygous c.238G>A and c.1534T>A	NM_003560
5	C–II 1	F/29yr	Chinese	Walk unstably	+	Yes–four limbs	–	Cerebellar signs, dysarthria, and intellectual disability	ND	+	+	Compound heterozygous c.967G>A and c.116G>A	NM_003560.2

## Methods

### Subjects

Five patients from three Chinese families with PLAN were recruited from the Peking University First Hospital, whose clinical presentations including medical history, physical examination, brain magnetic resonance imaging, as well as biological sample with peripheral blood were collected from all subjects. This study received ethical approval from Peking University First Hospital.

### Genetic Analysis

Genomic DNA was extracted *via* standard procedures from peripheral blood samples taken from all 5 patients. Sequence variants were detected by whole exome sequencing (10). All the exomes were sequenced by an Illumina (novaseq 6000) platform. Filtering strategies were the same as what were adopted in a previous study. Co-segregation analysis of the detected candidate variants were performed in their families (10). To predict the potential pathogenicity of genetic variants, *in silico* prediction analysis was performed in accordance with the guideline stipulated by the American college of medical genetics and genomics (ACMG) (11). *In silico* algorithms including Mutation Taster (http://www.mutationtaster.org) (12), PolyPhen-2 (http://genetics.bwh.harvard.edu/pph2/), and SIFT (http://sift.jcvi.org) (13) are applied.

### Literature Review

PubMed (03/20/2021) for ANAD and adult onset *PLA2G6*-related parkinsonism in genetically or neuropathology confirmed PLAN were systematically searched with the following terms: atypical neuroaxonal dystrophy, ANAD, PLAN, and parkinsonism. An in-depth review was carried out over all previously published case reports about ANAD and *PLA2G6* related and adult-onset parkinsonism, Only cases carrying biallelic PLA2G6 mutations with individual information were included. In parkinsonism type, childhood onset cases were excluded. Corresponding demographic, clinical, genetic, and radiological results were summarized as well.

## Results

### Case Descriptions

**Figure 1:**
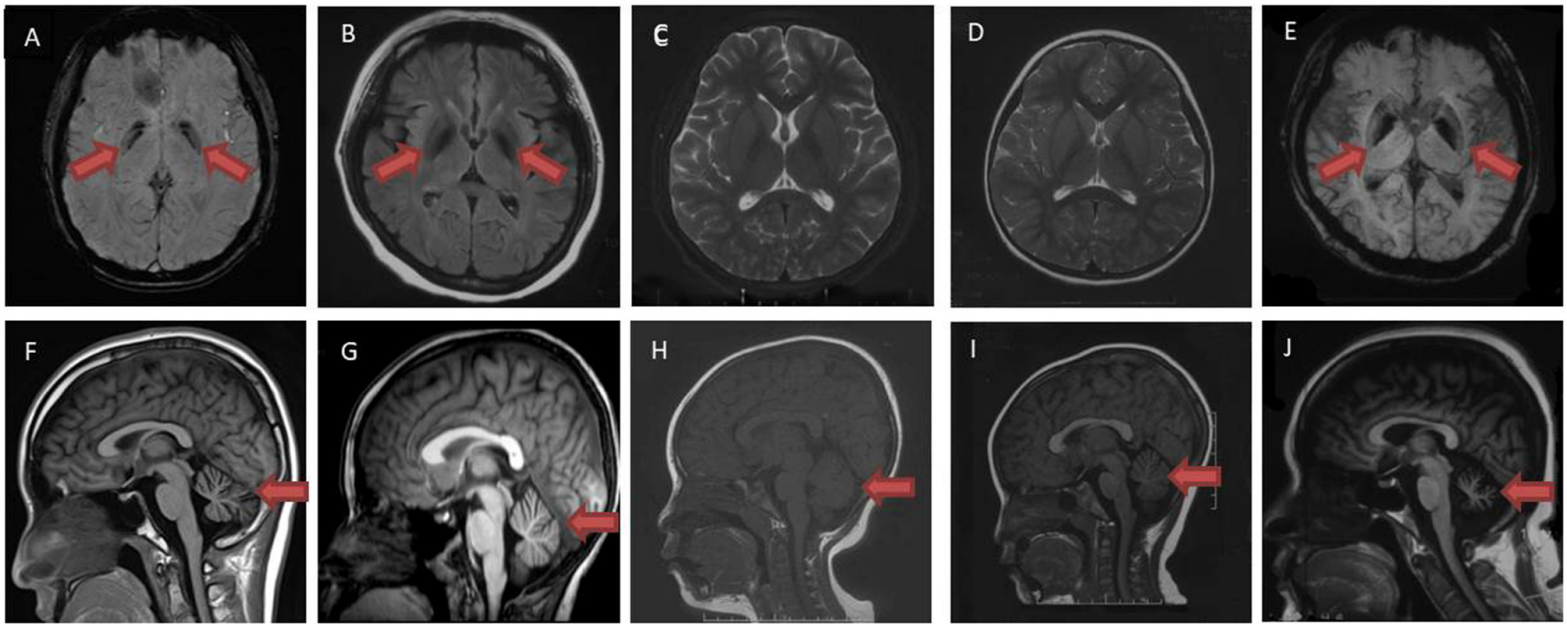
Brain magnetic resonance imaging examination of patient A II-1 **(A,F)**, A II-2 **(B,G)**, B-II 1 **(C,H)**, B-II 2 **(D,I)** and C-II 1 **(E,J)**. Susceptibility weighted imaging sequences **(A,E)** and T2 flair sequences **(B)** demonstrated iron deposition in bilateral globus pallidus (red arrow). No iron deposition was shown in T2 sequence **(C,D)** in patient B-II 1, B-II 2. T1 sequences demonstrated cerebellar atrophy in patient A II-1, A II-2, B-II 1, B-II 2, C-II 1 **(F–J)** (red arrow).

Patient A-II 1, the proband in family A, was a 14-year-old male who was asymptomatic until age 5, when he showed unstable gait without obvious weakness. Over the next 4 years, the symptoms progressed to rigidity in four limbs, and also caused decline in academic performance. At the age of 11, he could not walk by himself. Even the adoption of L-dopa failed to effectively improve these symptoms. Physical examinations revealed neck and trunk dystonia, dysarthria, and mental impairment. He got incomplete ophthalmoplegia. Muscle tone in both lower limbs were significantly increased such that he got spasticity. His incordination movements were impaired. Deep tendon reflexes were increased and bilateral Babinski's signs were positive. The maximum improvement rate of levodopa challenge test was 16.7%. Laboratory tests were normal, with no Kayser–Fleischer rings observed. Brain magnetic resonance imaging (Figure 1F) showed cerebellar atrophy; susceptibility weighted imaging sequencing (Figure 1A) demonstrated iron deposition in basal ganglia and substantia nigra.

Patient A-II 2 was an 8-year-old male who had ran slower than his peers since childhood. He walked slowly and could not run when he was 6, then he had difficulties squatting at the age of 7. Physical examinations showed his muscle tone in both lower limbs were significantly increased such that he got spasticity. His hand rotations were clumsy. In addition his deep tendon reflexes were increased. Bilateral Babinski's signs and Rossolimo's signs were positive. Brain magnetic resonance imaging (Figure 1G) showed cerebellar atrophy, and T2 Flair sequencing (Figure 1B) demonstrated iron deposition in basal ganglia and substantia nigra. The symptoms slightly improved after L-dopa administration.

Patient B-II 1, the proband in family B, was a 10-year-old female. Her four limbs became rigid and she had difficulties walking since age 3. When she was 5, she got dysarthria and mental decline, and she got dysphagia at 7. Physical examinations revealed dystonia in her face, neck, and hands. Her cognitive function was impaired. Muscle tone in lower limbs was increased. Deep tendon reflexes were increased in lower limbs. Left Babinski's sign was positive. Brain magnetic resonance imaging showed no obvious iron deposition in T2 sequence (Figure 1C), and cerebellar atrophy was specific (Figure 1H).

Patient B-II 2, the little sister of patient 3, was a 5-year-old female. When she was 2, she got dysarthria and mental decline. She walked in a weird posture at 3 years old and the symptoms progressed to rigidity in 4 limbs, which stopped her from walking at 4.5 years old. She and her elder sister felt no improvement after the administration of L-dopa. She got large amplitude dystonia in the trunk and neck during physical examination, but no dysphagia. Her cognitive function was impaired. Sensory examinations were normal. In lower limbs, muscle tone and deep tendon reflexes were increased. Bilateral Babinski's signs were positive. Brain magnetic resonance imaging when she was 3 years old showed no iron deposition in T2 sequence (Figure 1D), but there was cerebellar atrophy in T1 sequence (Figure 1I).

Patient C-II 1 was a 39 year-old female who was asymptomatic until 29 years old, when she began to walk unstably and felt weakness and rigidity in her lower limbs. The symptoms progressed to frequent falls and partly improved after the administration of L-dopa. Physical examinations revealed dysarthria and her cognitive functions were impaired. She got incomplete ophthalmoplegia. Muscle strength was decreased in her lower limbs, but was normal in upper limbs. Muscle tone in four limbs was increased, especially in upper limbs. Hand rotations were clumsy. Deep tendon reflexes in both lower limbs disappeared. Bilateral Babinski's signs were negative, while bilateral Chaddock signs were positive. Kayser–Fleischer rings were not observed, and ceruloplasmin was normal. Brain magnetic resonance imaging (Figure 1J) showed cerebellar atrophy, while susceptibility weighted imaging (Figure 1E) demonstrated iron deposition in bilateral globus pallidus. Electroencephalogram showed Paroxysmal slow waves in all leads. FDG PET/CT showed glucose metabolism was slightly reduced in her left temporal lobe and moderately to severely reduced in bilateral cerebellar hemisphere. Brain magnetic resonance imaging (Figure 1J) showed cerebellar atrophy, while susceptibility weighted imaging sequencing (Figure 1E) demonstrated iron deposition in basal ganglia and substantia nigra.

### Genetic Analysis

**Figure 2:**
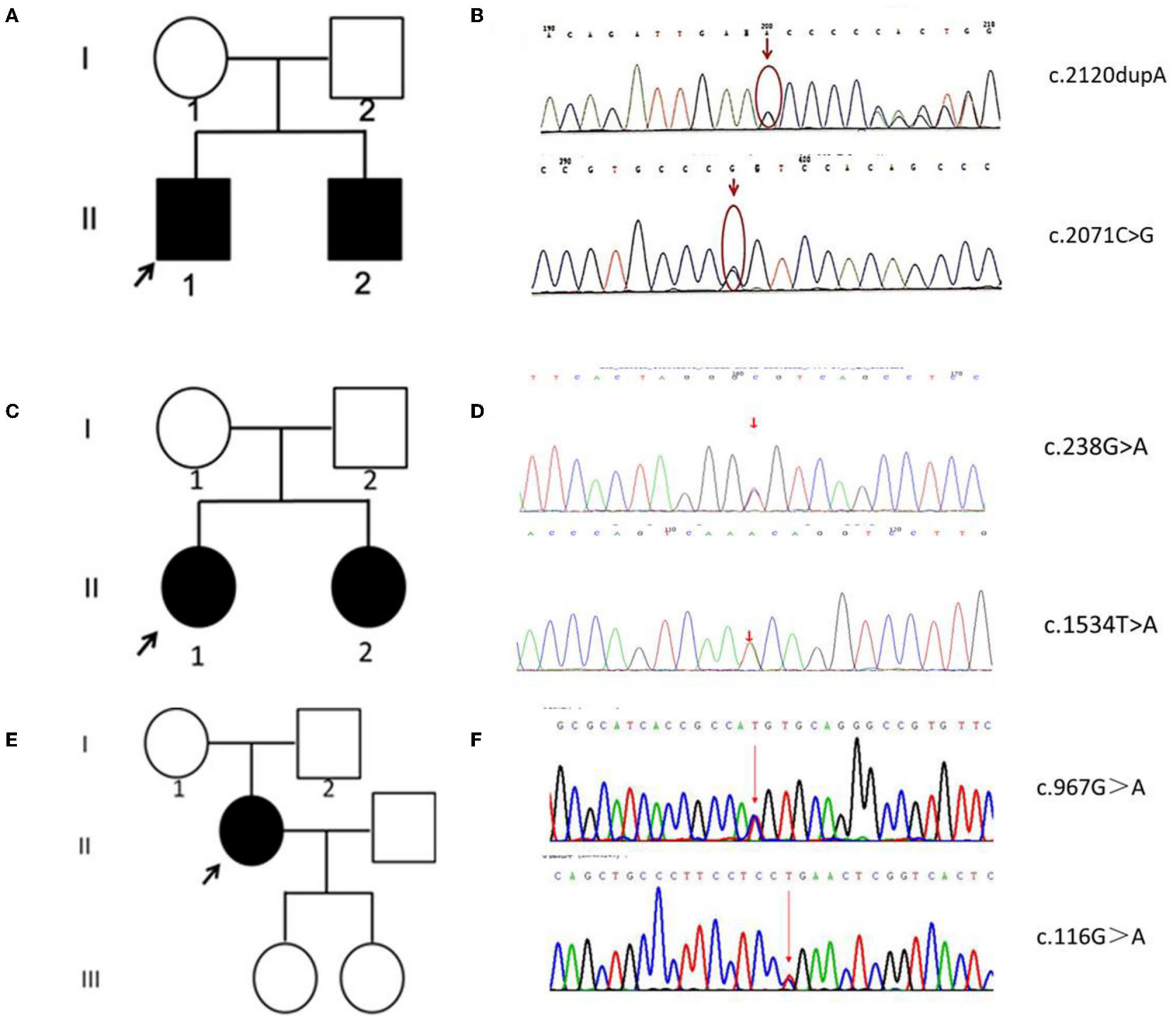
Family pedigrees and Sanger sequencing data of five patients. **(A)** The pedigree chart of family A. **(B)** The Sanger sequence chromatogram of family A. **(C)** The pedigree chart of family B. **(D)** The Sanger sequence chromatogram of family B. **(E)** The pedigree chart of family C. **(F)** The Sanger sequence chromatogram of family C. Open symbol: unaffected; filled symbol: affected; black arrows: proband. Red arrows: mutation sites.

In patient A-II 1 and patient A-II 2, the whole genome sequencing revealed compound heterozygous variants of c.2120dupA resulting in p.Asn707fs transition, and c.2071C>G resulting in p.Arg691Gly transition (Figures 2A,B). Their father and mother were proven heterozygous for c.2071C>G and c.2120dupA separately. The variant c.2120dupA and c.2071C>G have not been reported in ClinVar and Pubmed Database.

In patient B-II 1 and patient B-II 2, the whole genome sequencing revealed compound heterozygous variants of c.238G>A resulting in p.Ala80Thr transition, and c.1534T>A resulting in p.Phe512Ile (Figures 2C,D). Their father and mother were proven heterozygous for c.238G>A and c.1534T>A separately. The variant c.1534T>A has not been reported in ClinVar and Pubmed Database, while c.238G>A has been reported in INAD.

In patient C-II 1, the whole genome sequencing revealed compound heterozygous variants of c.967G>A resulting in p.Val323Met transition, and c.116G>A resulting in p.Arg39Gln transition (Figures 2E,F). Their father and mother were proven heterozygous for c.967G>A and c.116G>A separately. The variant c.967G>A has not been reported in ClinVar and Pubmed Database, while c.116G>A has been reported in INAD.

The predicting results of ACMG standard, Mutation Taster, and SIFT of the four novel sites were listed in the Supplementary Table.

### Literature Review

For ANAD, we screened 9 references for eligibility from PubMed search results (*n* = 3) and their reference lists. We found 7 references, and predefined data were extracted. The summary of the demographic, clinical, genetic, and radiological data of the reported 21 patients in literature (Table 2) (4, 5, 14–18) was shown in Table 1, where among the 21 ANAD patients, 86% (18/21) have iron deposition, 62% (13/21) parkinsonism, 71% (15/21) cerebellar atrophy, 67% (14/21) pyramidal signs, 52% (11/21) cognitive impairment, and 29% (6/21) nystagmus or Strabismus. Genetic analysis had been conducted on eleven of the reported ANAD patients, and c.238G>A was found to be the most popular mutation, which was detected in two unrelated pedigrees.

**Table 2:** Summary of demographic and clinical findings of reported 21 ANAD patients.

**Author, year**	**Patient**	**Ethniity/** **Reported country**	**Gender/ Age at Onset**	**Presenting Symptom**	**Pyramidal signs**	**Parkinsonism** **/Dystonia**	**Nystagmus / Strabismus**	**Other signs**	**Axonal spheroids**	**Cerebellar atrophy**	**Iron deposition**	**Genotype**
Nardocci N et al. (4), 1999	5	Italy	M/1yr	Slowing of psychomotor development with severe vomiting	–	–	+ (Strabismus)	Hypotonic–areflexictetraparesis, dementia	+	–	–	ND
	7	Italy	M/6mo	Slowing of psychomotor development and vision failure	–	–	–	Blindness, hypotonic–areflexictetraparesis, dementia, occasional generalized convulsive seizures after 6.	+	+	–	ND
	10	Italy	M/1.6yr	Mental impairment and gait disturbances	–	–	–	Hypotonic–areflexictetraparesis with cerebellar signs and dementia	–	+	+	ND
	11	Italy	M/2yr	Gait disturbances	–	–	+ (Nystagmus)	Hypotonic–areflexictetraparesis, cerebellar signs, dementia	+	+	+	ND
Salih MA et al. (14), 2013	F5 (P1)	Arabian	M/10yr	Not reported.	Brisk deep tendon reflexes	Bradykinesia	+	Heel cord tightening	ND	+	+	Homozygous c.2218G>A
Illingworth MA et al. (5), 2014	5	White Caucasian	F/3yr	Walk unstable	–	Dystonia and rigidity	–	Dysarthria	ND	–	+	Compound heterozygote c.2370T>G/ c.691G>C
Kapoor S et al. (15), 2016	4	Indian	F/2yr	Not reported	+	Dystonia	+	Not reported.	ND	+	+	ND
	5	Indian	F/2yr	Not reported	+	Dystonia	+	Not reported.	ND	–	+	Homozygous c.238G>A
	8	Indian	M/8yr	Not reported	+	Dystonia, bradykinesia	–	Not reported.	–	+	+	ND
	13	Indian	M/6yr	Not reported	+	Dystonia	–	Not reported.	–	–	+	ND
	15	Indian	M/2yr	Not reported	+	–	–	Developmental delay, early onset ataxia, slow saccades	ND	+	+	ND
	18	Indian	M/8yr	Not reported	+	Dystonia, choreiform movements	+	Ataxia, dystonia, choreiform movements	–	–	+	ND
	20	Indian	M/8yr	Not reported	+	–	–	Ataxia, action myoclonus	–	–	+	ND
	23	Indian	M/9yr	Not reported	+	Dystonia	–	Ataxia	–	+	+	Homozygous c.1946G>A
	24	Indian	F/8yr	Not reported	+	Generalized dystonia	–	Not reported.	ND	+	+	ND
Ma LM et al. (16), 2019	II3	Chinese	M/17yr	Fall down	+	Hypertonia	–	Cognitive impairment	ND	+	+	Compound heterozygous c.238G>A/ c.991G>T
	II1	Chinese	F/10yr	Walk unstable	+	Hypertonia	–	Cognitive impairment	ND	+	–	Compound heterozygous c.238G>A/ c.991G>T
Jain S et al. (17), 2019	P	Indian	F/7yr	Walk unstable	–	Hypertonia	–	Developmental delay	ND	+	+	Compound heterozygous c.1798C>T/ c.2357C>T
Toth–Bencsik R et al. (18)	1V/2	Hungarian	F/3yr	Gait instability	+	–	–	Mental deterioration, intention tremor	–	+	+	Compound heterozygous c.1864C >T/ c.1798C > T
	IV/3	Hungarian	F/3yr	Emotional lability	+	Hypertonia	–	Mental deterioration, intention tremor	ND	+	+	Compound heterozygous c.1864C >T/ c.1798C > T
	IV/4	Hungarian	F/2yr	Gait instability	+	–	–	Mental deterioration, intention tremor	ND	+	+	Compound heterozygous c.1864C >T/ c.1798C > T

*F, female; M, male; mo, month; yr, year; +, symptom present;-, symptom absent; ND, not done*.

For adult onset ANAD, we screened 24 references for eligibility from PubMed search results (*n* = 19) and their reference lists. The demographic, clinical, genetic, and radiological data of the 34 patients described in those articles (Table 3) (6, 8, 19–29) were summarized Table 3. Childhood onset cases and cases that didn't conform to the phenotype of AREP were excluded. Among the 34 adult onset PLA2G6-related parkinsonism patients, 76% (26/34) had pyramidal signs, 38% (13/34) displayed cerebellar atrophy, and 26% (9/34) iron deposition. Genetic analysis had been conducted on all of the 34 patients, and c. 2222G > A was found to be the most popular mutation. Eleven patients had c. 2222G > A homozygous mutation. Moreover, patients with homozygous 2222G > A mutation showed mostly neuropsychiatric symptoms as initial symptoms. And, inconsistent with previous reports (30), c.991G > T mutation was almost exclusively found in Chinese patients.

**Table 3:** Summary of demographic and clinical findings of reported 34 adult onset *PLA2G6*-related parkinsonism patients.

**Auther, year**	**Patient**	**Ethnicity /Reported country**	**Gender/ Age at Onset**	**Presenting Symptom**	**Pyramidal signs**	**Parkinsonism** **/Dystonia**	**Nystagmus / Strabismus**	**Other signs**	**Axonal spheroids**	**Cerebellar atrophy**	**Iron deposition**	**Genotype**
Paisan-Ruiz C et al. (6), 2009	F1P1	UK	F/26	Cognitive decline	+	+(with dystonia)	+	Psychiatric symptoms; levodopa-responsive; Dysarthria	ND	–	–	Homozygous c.2222G > A
	F2	UK	F/18	Foot drag	+	+(with dystonia)	+	Psychiatric symptoms; levodopa-responsive;	ND	–	–	Homozygous c.2239C > T
Sina F et al. (19), 2009	DP3	Iranian	M/25	Foot drag	+	+(with dystonia)	Not reported	Cognitive decline	ND	–	–	Homozygous c.1894C>T
	DP4	Iranian	M/22	Foot drag	+	+(with dystonia)	Not reported	Cognitive decline; Psychiatric features	ND	–	–	Homozygous c.1894C>T
	DP5	Iranian	F/21	Foot drag	+	+(with dystonia)	Not reported	Cognitive decline; Psychiatric features	ND	–	–	Homozygous c.1894C>T
Yoshino H, et al. (20), 2010	A	Japanese	F/20	Resting tremor, gait disturbance	+	+	Not reported	Depression; dementia; levodopa response	ND	–	+	Compound heterozygous c.216C>A/c. 1904G>A
	B1	Japanese	M/25	Gait disturbance	+	+	Not reported	Dementia; levodopa response	ND	–	–	Compound heterozygous c.1354C>T/c. 1904G>A
	B2	Japanese	M/30	Gait disturbance	–	+	Not reported	Levodopa response	ND	–	–	Compound heterozygous c.1354C>T/c. 1904G>A
Bower MA et al. (21), 2011	P	French, German, Irish, and English ancestry	F/18	Depression	+	+	Not reported	Hypophonia	ND	+	+	Compound heterozygous c.4C>A/ Del Ex 3
Virmani T et al. (22), 2014	P1	USA	F/25	Depression and psychosis	+	+	+	Dysphagia	ND	+	–	Homozygous c.2222G > A
	P2	USA	F/22	Depression	+	+	–	Cognitive deficit; tremor.	ND	+	–	Homozygous c.2222G > A
Malaguti MC et al. (23), 2015	P	Italian	F/27	Stiff leg sensation	+	+(with dystonia)	+	Mild dysarthria; dysphoric and anosognosic behavior	ND	–	+	Homozygous c.1547C>T
Xie F et al. (24), 2015	A	Chinese	M/36	Gait disturbance	–	+	Not reported	Mild resting tremor	ND	–	–	Homozygous c.991G > T mutations
	B	Chinese	M/36	Resting tremor	–	+	Not reported	Not reported	ND	–	–	Homozygous c.991G > T mutations
Kim YJ et al. (25), 2015	P1	Korean	F/22	Unsteady gait and falls	+	+(with dystonia)	Not reported	Dysarthria and microphonia	ND	+	+	Compound heterozygous c.1039G>A /c.1670C>T
Giri A et al. (26), 2016	P	Turkish	F/27	Left limb slowness	–	+	–	Hypomimia; hypophonia	ND	–	+	Homozygous c.2239C > T
Bohlega SA et al. (27), 2016	P1F1	Arabian	F/26	Depression, bradykinesia	+	+	Not reported	Levodopa response; autonomic symptoms	ND	–	–	Homozygous c.2222G > A
	P2F1	Arabian	M/22	Depression, tremor	+	+	Not reported	Levodopa response; autonomic symptoms	ND	–	–	Homozygous c.2222G > A
	P3F1	Arabian	M/23	Bradykinesia	+	+	Not reported	Levodopa response; autonomic symptoms	ND	–	–	Homozygous c.2222G > A
	P1F2	Arabian	F/25	Neuropsychiatric symptoms	+	+	Not reported	Levodopa response	ND	–	–	Homozygous c.2222G > A
Wirth T et al. (28), 2017	P1	Caucasian	M/23	Depression and anxiety	–	+(with dystonia)	Not reported	Severe psychiatric and behavioral disorders	ND	–	–	Compound heterozygous c.109C> T/ c.2321G > T
	P2	Caucasian	M/27	Left leg tremor and anxiety	+	+	Not reported	Psychiatric symptoms	ND	–	–	Compound heterozygous c.758G> T /c.2341G > A
Rohani M et al. (29), 2018	P	Arabian	M/18	Bradykinesia, tremor	+	+	Not reported	Levodopa response	ND	–	–	Homozygous p. Ala681Cysfs*92
Magrinelli F et al. (8), 2021	1	White British	F/27	Dystonia right arm	+	+(with dystonia)	Not reported	Cerebellar signs; myoclonus; cognitive impairment; anxiety depression.	ND	+	–	Compound heterozygote c.956C> T/c.1061T>C
	2	White British	F/29	Parkinsonism and executive dysfunction	+	+(with dystonia)	–	Cerebellar signs; myoclonus; cognitive; impairment; anxiety depression; apathy; urinary issues.	ND	+	+	Compound heterozygous c.238G>A /c.1924A>G
	3	Indian	F/21	Parkinsonism and psychiatric features	–	+(with dystonia)	–	Cerebellar signs; dysphagia; cognitive; impairment; anxiety; depression; urinary issues.	ND	+	+	Compound heterozygous c.673C>T /c.2311G>A
	6	Indian	M/29	Parkinsonism	–	+	–	Cognitive impairment; anxiety; depression; urinary issues; emotional lability; apathy.	ND	+	–	Homozygote c.1937C>T
	7	Indian	F/25	Parkinsonism	–	+	–	Cognitive impairment; depression; urinary issues; emotional lability; apathy; constipation.	ND	+	–	Compound heterozygote c.2370T> G/c.1511C>T
	9	Indian	F/22	Parkinsonism, dystonia, and behavioral issues	+	+(with dystonia)	–	Postural instability cognitive impairment apathy abusive behaviors emotional lability	ND	+	+	Homozygote c.2222G>A
	10	Pakistani	F/23	Psychiatric features	+	+	–	Dysarthria; myoclomus; cognitive impairment; anxiety; depression; emotional lability; urinary issues	ND	+	+	Homozygote c.2222G>A
	11	Pakistani	F/21	Psychiatric features	+	–	–	Cognitive impairment; myoclonus; anxiety; depression; emotional lability.	ND	–	–	Homozygote c.2222G>A
	12	German	M/22	Balance difficulty, bradykinesia	+	+	–	Cerebellar signs; dysarthria; cognitive impairment; dysphagia	ND	+	–	Compound heterozygote c.1021G>A /c.1898C>T
	13	Indian	M/21	Psychiatric features	+	+	–	Blepharospasm; pyramidal signs; cerebellar signs; dysarthria; cognitive impairment; behavioral issues	ND	+	–	Homozygote c.2222G>A
	14	Pakistani	M/31	Gait and balance difficulties	+	+	–	Cerebellar signs; myoclonus; cognitive impairment	ND	+	+	Homozygote c.2239C>T

## Discussion

*PLA2G6*-associated neurodegeneration (PLAN) is a heterogenous group of neurodegenerative diseases caused by mutations of *PLA2G6*. According to the onset age and clinical features, PLAN can be mainly classified into four subtypes, i.e., infantile onset INAD, childhood onset ANAD, adult-onset DP, and AREP. Clinically, PLAN belongs to complex movement disorders, since extrapyramidal manifestations (parkinsonism/dystonia) are shared by all subtypes, with the combination of psychomotor deterioration, ataxia, pyramidal signs, etc. In this study, four patients (Patient 1–4) presenting psychomotor decline, rigidity, ataxia, bradykinesia, dystonia, and autosomal recessive inheritance, with their onset ages between 2–6 years old, were clinically diagnosed as ANAD. The symptoms of Patient 5 were consistent with AREP based on adolescent-onset parkinsonism accompanied with pyramidal signs. Magnetic resonance imaging showed cerebellar atrophy and iron deposition in globus pallidus in all five patients. Finally next generation sequencing confirmed pathogenic variants in *PLA2G6*, and excluded other possible genetic causes. *PLA2G6* gene is located on chromosome 22q13.1, with 17 exons included. To date, the Human Gene Mutation Database (http://www.hgmd.cf.ac.uk/ac/gene.php?gene =PLA2G6) has reported a total of 238 *PLA2G6* mutations, including 178 missense mutations, 14 splicing-site mutations, 32 small indels, 11 gross deletions, and 4 gross insertions. The reported variants are widely distributed in the gene. Here, 6 variants of *PLA2G6* were identified, which included 4 novel and 2 known variants (https://preview.ncbi.nlm.nih.gov/clinvar/variation/437465). Co-segregation analysis on the parents of the patients confirmed all five patients carrying compound heterozygous mutations. Among the 4 novel variants, c.2120dupA was predicted to be pathogenic and c.2071C>G, c.967G>A, and c.1534T>A, to be likely pathogenic, in accordance with ACMG standards (11).

**Figure 3:**
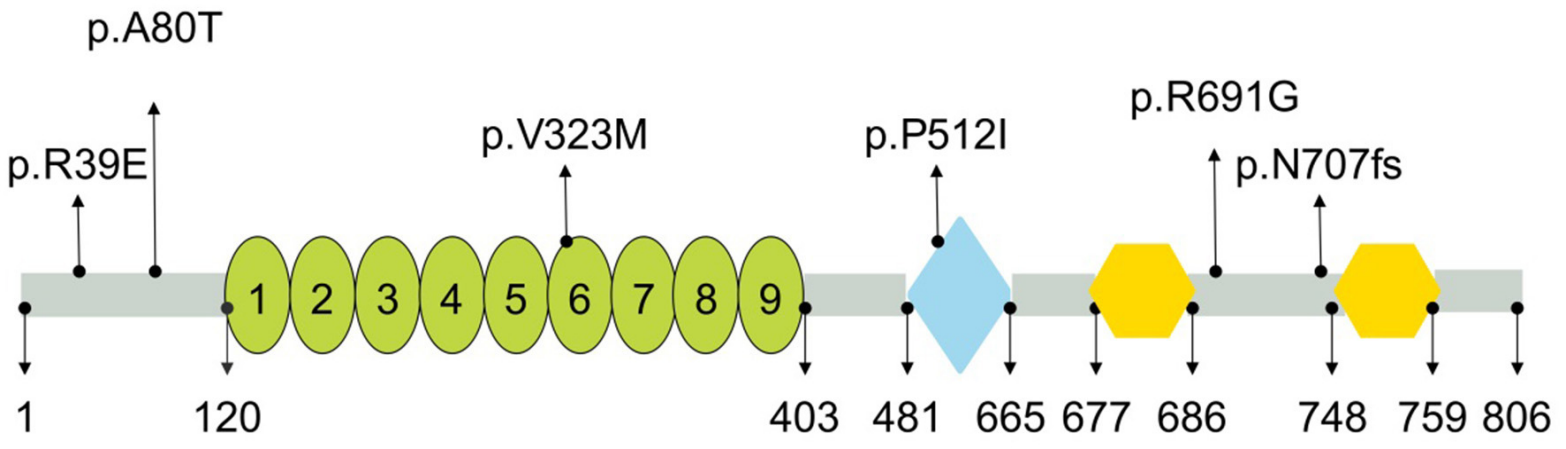
Schematic representation of PLA2G6 and location of mutations identified in present study. PLA2G6 consisted of nine ankyrin repeats (oval), patatin-like phospholipase (diamond), and two binding sites for calmodulin (hexagon). Numbers shown below were the amino acid positions (https://www.uniprot.org/).

*PLA2G6* gene encodes a protein named Ca2+-independent phospholipase A2 (iPLA2), an enzyme causing the release of free fatty acids and lysophospholipids by catalyzing the hydrolysis of the sn-2 fatty acyl bond of phospholipids (31), which plays an essential role in phospholipid remodeling, signal transduction, cell proliferation, endoplasmic reticulum stress-mediated apoptosis, and ferroptosis (32, 33). Multiple studies have verified that iPLA2 deficiency may alter membrane permeability, fluidity, and ion homeostasis, thereby causing mitochondrial abnormalities (24). The *PLA2G6* (iPLA2-VIA) protein harbors various domains from the N-terminus to C-terminus, including ankyrin repeats, patatin-like phospholipase, and two binding sites for calmodulin (https://www.uniprot.org/). Previous studies have also revealed that mutation sites in different domains may lead to different enzyme activities, which could be a key factor for the phenotypic variability (34). For example, mutations in the ankyrin repeat domain causes significant decreases in enzymatic activity, and results in INAD phenotype (34). In this study, there was only one mutation located in patatin-like phospholipase domain among the four mutation sites of the four ANAD patients, while the others were located in regions between domains; the simplex AREP patient had 1 mutation in the ankyrin repeats, and another one was in regions between domains (Figure 3), which was in line with results of other studies. Most of the mutations reported in these studies were not located in domains, which may explain the reason why the patients in question presented milder clinical features than INAD.

The case series in this study highlighted the classical, atypical clinical, and neuroimaging features of *PLA2G6* gene related ANAD/AREP. A review of all ANAD patients reported so far showed that the most common features of ANAD were iron deposition of globus pallidus, cerebellar atrophy, pyramidal signs, and parkinsonism, accounting for 86, 71, 67, and 62%, respectively, which also appeared in four ANAD patients in this study. Notably, all of the four ANAD patients in question presented prominent spastic rigidity in lower limbs in their early stage, making them misdiagnosed as complex hereditary spastic paraplegia (HSP) for a long time. Burcak Ozes et al. reported two affected Turkish siblings presenting HSP with *PLA2G6* c.2239C>T homozygous missense (35). And C19ORF12 (*SPG43*) gene was also reported to be responsible for both HSP and NBIA (36). However, patients in question also showed significant cerebellar signs, cognitive impairment, parkinsonism, and dystonia, indicating they are different from those suffering from pure HSP. Importantly, imaging showed 3 of the 5 patients had iron deposition and cerebellar atrophy, which was consistent with the literature where 86% reported ANAD cases got iron deposition. As the disease developed, the iron deposition and cerebellar atrophy became severe, when physicians were reminded to follow up magnetic resonance imaging when considering the diagnosis of PLAN. However, inconsistent with literature review, c.238G>A was proven to be the most popular mutation in ANAD patients and had been reported in two Chinese patients and one Indian patient.

Literature review of *PLA2G6*-AREP patients showed that, in addition to early-onset parkinsonism, pyramidal signs (76%), cerebellar atrophy (38%), and iron deposition of globus pallidus (26%) are also common in these patients. Different from those with ANAD, the present Patient 5, who was sporadic, showed dopa-responsive parkinsonism with additional pyramidal signs. Neither of the mutations had been reported in *PLA2G6*-related parkinsonism, making it hard to distinguish AREP from other early onset genetic parkinsonism only by these clinical features. Again, iron deposition and cerebellar atrophy on magnetic resonance imaging could give helpful clues to the underlying genetic causes, while NGS could help make a definite diagnosis.

In conclusion, great clinical heterogeneity was shown in *PLA2G6*-neurodegeneration, making these patients prone to misdiagnosis or delay diagnosis. Brain magnetic resonance imaging could provide helpful diagnostic clues and next generation sequencing played an important role in diagnosis. The four novel pathogenic variants (c.2120dupA, c.2071C>G, c.967G>A, c.1534T>A) identified in this study enriched the mutational spectrum of *PLA2G6*-associated neurodegeneration.

## Data Availability Statement

The datasets which support the findings of the present article are included in the manuscript/supplementary material. Queries should be directed to the corresponding author(s).

## Ethics Statement

The studies involving human participants were reviewed and approved by the Peking University First Hospital Ethics Committee. Written informed consent to participate in this study was provided by the participants' legal guardian/next of kin.

## Author Contributions

Study conception and design: YW, YJ, RL, ZW, YY, WS, and HJ. Analysis and interpretation of results: YW, ZX, CL, and KD. Draft manuscript preparation: YW, HJ, CL, ZX, and WS. All authors reviewed the results and approved the final version of the manuscript.

## Funding

This work was supported by the National Natural Science Foundation of China [Grant Number 82071306].

## Conflict of Interest

The authors declare that the research was conducted in the absence of any commercial or financial relationships that could be construed as a potential conflict of interest.

## Publisher's Note

All claims expressed in this article are solely those of the authors and do not necessarily represent those of their affiliated organizations, or those of the publisher, the editors and the reviewers. Any product that may be evaluated in this article, or claim that may be made by its manufacturer, is not guaranteed or endorsed by the publisher.
