# Supplementary material for: Novel PLA2G6 Pathogenic Variants in Chinese Patients With PLA2G6-Associated Neurodegeneration
Source: Front Neurol. 2022 Jul 13;13:922528. doi: 10.3389/fneur.2022.922528 (PMC9327523; doi:10.3389/fneur.2022.922528)
Supplement: Supplementary file 1 [file Table_1.DOCX]

Supplementary Table 1 In silico analysis of four novel variants in PLAN.

|  | ACMG | Mutation  taster | Polyphen-2 | SIFT |
| --- | --- | --- | --- | --- |
| c.2120dupA | Pathogenic  (PVS1+PM2+PP4) |  |  |  |
| c.2071C＞G | Likely Pathogenic  (PM2+PM3+PP3+PP4) | Damaging | Probably damaging | Damaging |
| c.967G＞A | Uncertain significance  (PM2+PM3+PP3+PP4) | Damaging | Probably damaging | Damaging |
| c.1534T＞A | Likely pathogenic  (PM3+PM2+PP3+PP4) | Damaging | Probably damaging | Damaging |

PolyPhen-2, polymorphism phenotyping 2; SIFT, sorting intolerant from tolerant;
